# Transcranial direct current stimulation for pain threshold in knee osteoarthritis: a mechanism-oriented systematic review and meta-analysis of randomized controlled trials

**DOI:** 10.3389/fphys.2026.1890510

**Published:** 2026-07-14

**Authors:** Yi Shangguan, Ziliang Zhou, Kai Liu, Zhengtong Qiao, Wenxin Xu

**Affiliations:** 1 Department of Rehabilitation Medicine, Qingdao Hospital, University of Health and Rehabilitation Sciences (Qingdao Municipal Hospital), Qingdao, China; 2 Rehabilitation Therapy Center, Luoyang Orthopedic-Traumatological Hospital (Henan Provincial Orthopedic Hospital), Luoyang, China

**Keywords:** knee osteoarthritis, meta-analysis, pain sensitivity, pain threshold, transcranial direct current stimulation

## Abstract

**Background:**

Knee osteoarthritis (KOA) pain is increasingly recognized to involve altered central pain processing, including central sensitization and impaired descending inhibitory pathways. Transcranial direct current stimulation (tDCS) may alleviate pain by modulating cortical excitability and pain-related networks. However, evidence on pain threshold remains fragmented.

**Objective:**

To systematically evaluate the effects of tDCS on pain threshold in patients with KOA, and to assess its effects on pain intensity, physical function, walking capacity, and safety.

**Methods:**

This systematic review and meta-analysis followed PRISMA 2020. MEDLINE, Embase, Cochrane Library, and Web of Science were searched from inception to October 7, 2025 for randomized controlled trials (RCTs) involving patients with KOA. Primary outcomes consisted of pain thresholds that reflect pain sensitivity, namely pressure pain threshold (PPT), conditioned pain modulation (CPM), heat pain threshold (HPTh), and heat pain tolerance (HPTo). Secondary outcomes included pain intensity (VAS/NRS), physical function (WOMAC/KOOS), and walking capacity (6MWT/10MWT). Risk of bias was assessed using RoB 2.0.

**Results:**

Seventeen RCTs involving 1,113 participants were included. Compared with controls, tDCS significantly improved pain threshold overall (SMD = 0.55, 95% CI: 0.32 to 0.78; *P* < 0.01; I² = 66.2%). Subgroup analyses showed significant improvements in CPM (SMD = 0.47, 95% CI: 0.18 to 0.77), HPTo (SMD = 0.40, 95% CI: 0.09 to 0.71), and PPT (SMD = 0.77, 95% CI: 0.25 to 1.30), whereas HPTh did not differ significantly from controls. tDCS also significantly reduced pain intensity (SMD = -0.65, 95% CI: -0.91 to -0.39; *P* < 0.01; I² = 67.3%). Physical function was improved (SMD = -0.38, 95% CI: -0.54 to -0.21, *P* < 0.01; I² = 0.0%). Risk of bias was rated as low in 9 studies, some concerns in 7 studies, and high in 1 study. Adverse events were generally mild and transient, with no serious intervention-related adverse events reported.

**Conclusions:**

Current evidence suggests that tDCS may improve pain threshold and reduce pain intensity in patients with KOA. However, the effects on physical function remain less certain because of substantial heterogeneity and potential risk-of-bias effects. Future high-quality RCTs with mechanistic stratification and standardized stimulation and assessment protocols are warranted.

**Systematic review registration:**

https://www.crd.york.ac.uk/prospero/display_record.php?, identifier CRD420261297687.

## 1 Introduction

Knee osteoarthritis (KOA) is a chronic degenerative disorder primarily characterized by articular cartilage degeneration and osteophyte formation. Clinically, it manifests as knee pain, stiffness, and limited mobility, which may progress to substantial functional impairment and disability in severe cases (Gelber, 2024). Globally, the prevalence of KOA among adults aged 40 years and older is 23%, affecting an estimated 654 million individuals (Cui et al., 2020). The lifetime risk of symptomatic knee osteoarthritis reaches 45% by the age of 85 years (Duong et al., 2023). Current management of KOA mainly consists of health education, exercise-based rehabilitation, and analgesic or anti-inflammatory medications. However, therapeutic responses vary considerably across individuals, and overall efficacy remains suboptimal (Bannuru et al., 2019; Moseng et al., 2024).

Pain in KOA is not strictly correlated with local joint pathological changes, indicating the involvement of aberrant central pain processing and modulation, such as central sensitization and impaired descending inhibitory pathways (Aoyagi et al., 2022). Consistently, Satake et al. reported that elevated pain sensitivity at the knee was associated with non–weight-bearing pain (Satake et al., 2021; Aoyagi et al., 2022). These findings suggest that pain sensitization (i.e., facilitation of ascending nociceptive pathways) may substantially contribute to pain experience in patients with KOA (Arant et al., 2022). Such central mechanisms may attenuate the efficacy of conventional peripheral-targeted treatments and underscore the need to explore novel therapeutic strategies targeting central pain modulation (Arant et al., 2022; Petersen et al., 2023).

Transcranial direct current stimulation (tDCS) is a non-invasive brain stimulation technique that modulates cortical excitability and regulates pain-related neural network activity through weak direct electrical currents (Lloyd et al., 2020). Dehghani et al. proposed that tDCS may enhance descending pain inhibition and reduce pain sensitivity, thereby alleviating pain in individuals with KOA (Dehghani et al., 2025; Rodríguez-Lagos et al., 2025). Compared with pharmacological and invasive interventions, tDCS offers several advantages, including favorable safety, repeatability, and compatibility with rehabilitation programs (Lloyd et al., 2020). Nevertheless, most existing studies on tDCS for KOA have used subjective pain scores as primary outcomes. Mechanistic outcomes such as pain threshold have been inconsistently reported, and high-quality clinical evidence remains limited (Lawford et al., 2024).

Against this background, we conducted this systematic review and meta-analysis to quantitatively evaluate the mechanistic effects of tDCS in patients with KOA. Pain sensitivity-related parameters served as the primary outcomes. In addition, we assessed the effects of tDCS on clinical pain intensity and knee function.

## 2 Methods

This systematic review and meta-analysis was conducted in adherence with the Preferred Reporting Items for Systematic Reviews and Meta-Analyses (PRISMA) 2020 statement, and was registered in the International Prospective Register of Systematic Reviews (PROSPERO) under the registration number CRD420261297687.

### 2.1 Search strategy

The systematic literature search was performed across four electronic databases, including MEDLINE, Embase, Cochrane Library, and Web of Science, from their respective inception dates to October 7, 2025. The search strategy was constructed using a combination of the key terms “tDCS” and “KOA”, together with their corresponding MeSH terms and free-text synonyms. The detailed full search strategy for each database is presented in the **Supplementary File**.

### 2.2 Inclusion/exclusion criteria

Eligibility criteria for study inclusion and exclusion were pre-specified *a priori* based on the Population, Intervention, Comparison, Outcome, and Study design (PICOS) research framework (Liberati et al., 2009). (1) Participants: Adult human subjects with a confirmed clinical diagnosis of KOA. (2) Intervention: Studies in which tDCS was administered as the primary therapeutic intervention; concurrent combination of tDCS with other conventional KOA treatments was permitted. (3) Comparator: Sham tDCS or any other standardized control intervention. (4) Outcomes: Primary and secondary outcomes included pain threshold and clinical knee assessment scales. (5) Study design: Randomized controlled trials (RCTs).

Studies were excluded if they met any of the following criteria: (1) were secondary research studies that synthesized findings from multiple primary RCTs; (2) were study protocols, abstracts, conference proceedings, or unpublished grey literature with no full-text data available; (3) reported incomplete or insufficient raw data; (4) were non-randomized controlled trials or observational studies.

### 2.3 Selection process

YSG and ZLZ independently screened the titles and abstracts of all retrieved articles, followed by full-text screening according to the pre-specified eligibility criteria. Duplicate studies were removed using EndNote X9 software combined with manual checking. Disagreements regarding the eligibility of studies were resolved through discussion to reach a consensus; when necessary, a third researcher was invited to act as an arbitrator.

### 2.4 Risk of bias

Risk of bias in the included studies was assessed using the Cochrane Risk of Bias Tool (RoB 2.0) (Brignardello-Petersen et al., 2018; Sterne et al., 2019), encompassing key domains such as the randomization process, deviations from intended interventions, missing outcome data, outcome measurement, and other potential sources of bias. A study was rated as having low risk of bias if all domains were judged as low. If one or more domains raised some concerns (e.g., unclear or insufficient information), the study was classified as having some concerns. Studies with one or more domains judged as high risk were considered to have high risk of bias. Two reviewers (YSG and ZLZ) independently conducted the assessments, and any disagreements were resolved by a third reviewer.

### 2.5 Data extraction

Data were extracted independently by two reviewers (YSG and ZLZ) and included basic study information (author, year, country), clinical characteristics (sample size, age, KOA type, time window), and intervention details (frequency, dose, training content, control conditions). Extracted outcomes comprised pain threshold measures (pressure pain threshold, PPT; conditioned pain modulation, CPM; heat pain threshold, HPTh; heat pain tolerance, HPTo), pain intensity (visual analog scale, VAS; numeric rating scale, NRS), physical function (Knee Injury and Osteoarthritis Outcome Score, KOOS; Western Ontario and McMaster Universities Osteoarthritis Index, WOMAC), and walking capacity (six-minute walk test, 6MWT; 10-Meter Walk Test, 10MWT). Data at baseline and at the latest follow-up after intervention were included for analysis. Additional data were obtained using image digitization software or by contacting the authors when necessary.

### 2.6 Statistical analysis

All meta-analyses were performed using Stata/MP 18.0 software. For the same outcome assessed by different measurement scales across studies, the standardized mean difference (SMD) with a 95% confidence interval (CI) was calculated. A random-effects model was adopted for primary analyses to account for potential clinical and methodological heterogeneity across included studies. The robustness of pooled results was verified using the leave-one-out sensitivity analysis. Statistical heterogeneity was evaluated by the I² statistic, with the following cut-offs: 0–25% (no heterogeneity), 26–50% (low heterogeneity), 51–75% (moderate heterogeneity), and >75% (high heterogeneity). Sensitivity analyses were conducted to explore potential sources of between-study heterogeneity, and subgroup analyses were performed for pain threshold outcomes to identify determinants of treatment effects (Higgins et al., 2003). Publication bias was assessed by Egger’s test and visual inspection of funnel plots. Sensitivity analyses excluding high-risk studies were conducted to examine the robustness of pooled estimates. All statistical tests were two-tailed, with statistical significance set at *P* < 0.05.

## 3 Results

### 3.1 Search results

**Figure 1:**
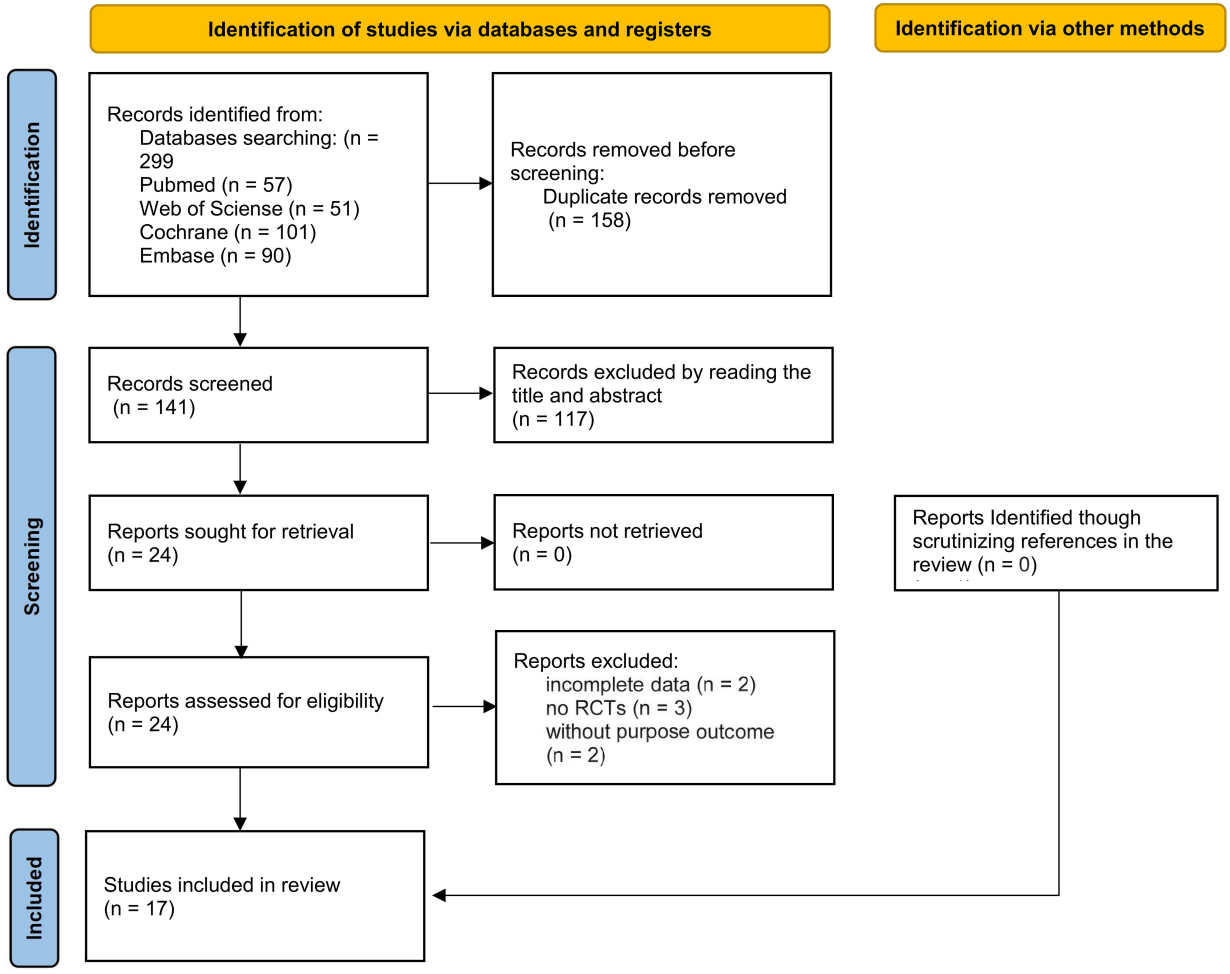
Flow diagram of the studies screened and included according to PRISMA.

An initial search identified 299 articles. After removing duplicates, 141 records remained. Screening of titles and abstracts resulted in the exclusion of 117 articles, leaving 24 articles for full-text review. Of these 24 articles, 2 were excluded due to incomplete data, 3 for outcome measures that did not meet the prespecified criteria, and 2 because they were not RCTs. Ultimately, 17 studies met all the inclusion criteria. A review of the reference lists of the included studies did not identify any additional relevant studies. The detailed study selection process is illustrated in **Figure 1**.

### 3.2 Study characteristics

A total of 17 studies involving 1,113 participants were included in this meta-analysis (Ahn et al., 2017; Chang et al., 2017; Ahn et al., 2018; Ahn et al., 2019; da Graca-Tarragó et al., 2019; Sajadi et al., 2020; Azizi et al., 2021; Rahimi et al., 2021; Tavares et al., 2021; Martorella et al., 2022a; Martorella et al., 2022b; Montilla-Herrador et al., 2024; Boonprasit et al., 2025; Kalkhoran and Khanmohammadi, 2025; Nie et al., 2025; Xia et al., 2025a; Xia et al., 2025b). These studies were mainly conducted in the United States (5 studies (Ahn et al., 2017; Ahn et al., 2018; Ahn et al., 2019; Martorella et al., 2022a; Martorella et al., 2022b); n=350), Iran (4 studies (Sajadi et al., 2020; Azizi et al., 2021; Rahimi et al., 2021; Kalkhoran and Khanmohammadi, 2025); n=242), China (3 studies (Nie et al., 2025; Xia et al., 2025a; Xia et al., 2025b); n=230), Brazil (2 studies (da Graca-Tarragó et al., 2019; Tavares et al., 2021); n=164), Spain (1 study (Montilla-Herrador et al., 2024); n=65), Thailand (1 study (Boonprasit et al., 2025); n=32), and Australia (1 study (Chang et al., 2017); n=30). Regarding gender distribution, among the 1,113 participants with extractable gender data, 828 (74.4%) were female and 285 (25.6%) were male; two studies (da Graca-Tarragó et al., 2019; Kalkhoran and Khanmohammadi, 2025) included only female participants. For diagnostic criteria, 11 studies (Ahn et al., 2017; Chang et al., 2017; Ahn et al., 2018; Ahn et al., 2019; da Graca-Tarragó et al., 2019; Sajadi et al., 2020; Rahimi et al., 2021; Tavares et al., 2021; Martorella et al., 2022b; Boonprasit et al., 2025) enrolled KOA patients based on the ACR criteria, 1 study (Montilla-Herrador et al., 2024) used the OARSI criteria, and the remaining 4 studies (Azizi et al., 2021; Martorella et al., 2022a; Nie et al., 2025; Xia et al., 2025a) did not explicitly specify the ACR criteria but reported the Kellgren–Lawrence (KL) grade as an inclusion condition. In addition, 1 study (Xia et al., 2025b) focused on a special population of KOA patients after stroke. Thirteen studies (Ahn et al., 2017; Chang et al., 2017; da Graca-Tarragó et al., 2019; Sajadi et al., 2020; Azizi et al., 2021; Rahimi et al., 2021; Tavares et al., 2021; Martorella et al., 2022a; Martorella et al., 2022b; Montilla-Herrador et al., 2024; Boonprasit et al., 2025; Nie et al., 2025; Xia et al., 2025a) reported the KL grade. Seven studies (Chang et al., 2017; Ahn et al., 2018; Ahn et al., 2019; da Graca-Tarragó et al., 2019; Tavares et al., 2021; Martorella et al., 2022a; Montilla-Herrador et al., 2024) described changes in pain threshold. Fourteen studies (Ahn et al., 2017; Chang et al., 2017; Ahn et al., 2019; da Graca-Tarragó et al., 2019; Sajadi et al., 2020; Azizi et al., 2021; Rahimi et al., 2021; Tavares et al., 2021; Martorella et al., 2022b; Montilla-Herrador et al., 2024; Boonprasit et al., 2025; Nie et al., 2025; Xia et al., 2025a; Xia et al., 2025b) reported pain intensity measured by the VAS or NRS. Twelve studies (Ahn et al., 2017; Chang et al., 2017; Ahn et al., 2019; da Graca-Tarragó et al., 2019; Sajadi et al., 2020; Azizi et al., 2021; Rahimi et al., 2021; Tavares et al., 2021; Martorella et al., 2022b; Montilla-Herrador et al., 2024; Boonprasit et al., 2025; Nie et al., 2025) described changes in physical function assessed by the KOOS and WOMAC. Six studies (Ahn et al., 2017; Rahimi et al., 2021; Montilla-Herrador et al., 2024; Kalkhoran and Khanmohammadi, 2025; Xia et al., 2025a; Xia et al., 2025b) reported walking ability evaluated by 6MWT and 10MWT. Detailed information is shown in **Table 1**.

**Table 1:** Summary of study characteristics.

Study	Country	KOA grade	Intervention/comparator (compressed)	Participants (n)	Sex (M/F)	Participants (Age)	tDCS (dose & montage)	Frequency	Follow-up	Key outcomes
(Nie et al., 2025)	China	KL Grade 0-2	Acupuncture + PT tDCS + PT Acupuncture + tDCS + PT	20 20 20	7/13 4/16 4/16	56.55 ± 8.00 56.15 ± 7.91 58.80 ± 7.06	2 mA/20 min; Anode: Contralateral M1 (C3/C4); Cathode: Contralateral supraorbital	2 weeks 5 sessions/week	–	VAS; WOMAC
(Xia et al., 2025a)	China	KL Grade 1-2	TENS +tDCS TENS + sham tDCS	55 55	23/32 15/40	64.96 ± 2.77 65.31 ± 3.09	2 mA/20 min; Anode: Cz; Cathode: Non-dominant supraorbital	4 weeks 5 sessions/week	2 months	VAS; 6MWT
(Kalkhoran and Khanmohammadi, 2025)	Iran	KL Grade ≥1	aquatic therapy + sham tDCS aquatic therapy + tDCS tDCS; sham tDCS	17 17 17 17	F only	65.05 ± 1.34 65.00 ± 1.50 65.47 ± 1.62 65.17 ± 1.66	2 mA/30 min; Anode: M1 (C3) + Right PPC (P4); Cathode: Fp1/Fp2	8 weeks 2 sessions/week	–	10MWT
(Azizi et al., 2021)	Iran	KL Grade 1-2	tDCS + Acetaminophen sham tDCS + Acetaminophen	27 27	6/21 9/18	61.3 ± 13.5 56.4 ± 11.7	2 mA/20 min; Anode: Contralateral M1 (C3/C4); Cathode: Ipsilateral supraorbital	1 week 5 sessions	3 months	VAS; KOOS
(Ahn et al., 2019)	USA	KOA (ACR Criteria)	tDCS + mindfulness sham tDCS + sham mindfulness	15 15	7/8 5/10	59.47 ± 6.91	2 mA/20 min; Anode: Contralateral M1; Cathode: Ipsilateral supraorbital	2 weeks 5 sessions/week	–	NRS; WOMAC; PPT; CPM
(Rahimi et al., 2021)	Iran	KL Grade 2-3	tDCS (M1) + PT tDCS (S1) + PT tDCS (DLPFC) + PT sham tDCS + PT	20 20 20 20	8/72 8/72	58.8 ± 3.3	1 mA/20 min; Anode: Left M1/S1/DLPFC; Cathode: Contralateral supraorbital	2 weeks 5 sessions/week	1 month	VAS; KOOS; 10MWT
(Ahn et al., 2017)	USA	KL Grade 0-4	tDCS sham tDCS	20 20	10/10 9/11	60.6 ± 9.8 59.3 ± 8.6	2 mA/20 min; Anode: Contralateral M1 (C3/C4); Cathode: Ipsilateral supraorbital	1 week 5 sessions/week	3 weeks	NRS; WOMAC; 6MWT
(Xia et al., 2025b)	China	Post-stroke KOA	tDCS + TENS sham tDCS + TENS	30 30	15/15 15/15	65.31 ± 3.09 63.70 ± 5.47	2mA/20min; Anode: Affected C3; Cathode: Contralateral supraorbital	8 weeks 5 sessions/week	–	VAS; 6MWT
(Sajadi et al., 2020)	Iran	KL Grade 2-3	TENS tDCS	20 20	5/15 1/19	59.30 ± 6.13 56.85 ± 5.81	2 mA/20 min; Anode: Contralateral M1 (C3/C4); Cathode: Ipsilateral supraorbital	2 weeks 3 sessions/week	3 months	VAS; WOMAC
(Tavares et al., 2021)	Brazil	KL Grade 2-4	tDCS sham tDCS	51 53	9/42 7/46	74.78 ± 7.44 73.13 ± 8.51	2 mA/20 min; Anode: Contralateral M1 (C3/C4); Cathode: Contralateral supraorbital	3 weeks 1 sessions/d, 15 sessions	2 months	VAS; WOMAC; PPT; CPM
(Chang et al., 2017)	Australia	KOA (ACR Criteria)	tDCS sham tDCS	15 15	4/11 6/9	59.8 ± 9.1 64.1 ± 11.1	2 mA/20 min; Anode: Contralateral M1; Cathode: Contralateral supraorbital	8 weeks 2 sessions/week	–	VAS; WOMAC; PPT;
(Ahn et al., 2018)	USA	KOA (ACR Criteria)	tDCS sham tDCS	20 20	10/10 9/11	60.6 ± 9.8 59.3 ± 8.6	2 mA/20 min; Anode: Contralateral M1 (C3/C4); Cathode: Contralateral supraorbital	1 week 5 sessions	–	NRS; PPT; CPM; HPTh; HPTo
(Martorella et al., 2022a)	USA	KOA (ACR Criteria)	tDCS sham tDCS	60 60	20/40 18/42	65.3 ± 8.4 66.6 ± 8.4	2 mA/20 min; Anode: Contralateral M1 (C3/C4); Cathode: Contralateral supraorbital	3 weeks 5 sessions/week	–	PPT; CPM; HPTh; HPTo
(Martorella et al., 2022b)	USA	KL Grade 1-4	tDCS sham tDCS	60 60	20/40 18/42	65.32 ± 8.41 66.60 ± 8.43	2 mA/20 min; Anode: Contralateral M1; Cathode: Contralateral supraorbital	3 weeks 5 sessions/week	3 months	NRS; WOMAC
(da Graca-Tarragó et al., 2019)	Brazil	KL Grade 3-4	tDCS + EIMS tDCS + shamEIMS sham tDCS + EIMS sham tDCS + shamEIMS	15 15 15 15	F only	50-75	2 mA/30 min; Anode: Contralateral M1; Cathode: Contralateral supraorbital	1 week 5 sessions/week	–	VAS; CPM; PPT; WOMAC
(Boonprasit et al., 2025)	Thailand	KL Grade 1-3	tDCS sham tDCS	16 16	1/15 2/14	64.44 ± 5.56 65.50 ± 7.06	2 mA/20 min; Anode: Left DLPFC (F3); Cathode: Right supraorbital	4 weeks 3 sessions/week	4 weeks	VAS; WOMAC
(Montilla-Herrador et al., 2024)	Spain	KL Grade 1-4	tDCS + TENS tDCS + shamTENS sham tDCS + shamTENS	20 24 21	18/47	68.05 ± 8.65	1.5 mA/20 min; Anode: Contralateral M1; Cathode: Ipsilateral supraorbital	2 weeks 5 sessions/week	6 months	VAS; WOMAC; 6MWT; 10MWT

KOA, Knee Osteoarthritis; KL Grade, Kellgren-Lawrence Grade; tDCS, Transcranial Direct Current Stimulation; VAS, Visual Analog Scale; WOMAC, Western Ontario and McMaster Universities Osteoarthritis Index; NRS, Numerical Rating Scale; 6MWT, 6-Minute Walk Test; 10MWT, 10-Meter Walk Test; PPT, Pressure Pain Threshold; CPM, Conditioned Pain Modulation; HPTh, Heat Pain Threshold; HPTo, Heat Pain Tolerance; EIMS, Electrical Intramuscular Stimulation; TENS, Transcutaneous Electrical Nerve Stimulation; ACR, American College of Rheumatology; M1, Primary Motor Cortex; S1, Primary Somatosensory Cortex; DLPFC, Dorsolateral Prefrontal Cortex; PPC, Posterior Parietal Cortex.

### 3.3 Pain threshold

A total of 7 studies were included for the analysis of pain threshold, with higher SMD values indicating an elevated pain threshold. The pooled results of the random-effects model showed that tDCS intervention significantly increased the pain threshold compared with the control group (SMD = 0.55, 95% CI: 0.32 to 0.78, *P* < 0.01), with moderate between-study heterogeneity (I² = 66.2%). Egger’s test and funnel plot suggested no significant publication bias (*P* = 0.754) (**Supplementary Figure 1**). Leave-one-out sensitivity analysis confirmed the robustness of the pooled effect size (**Supplementary Figure 2**).

**Figure 2:**
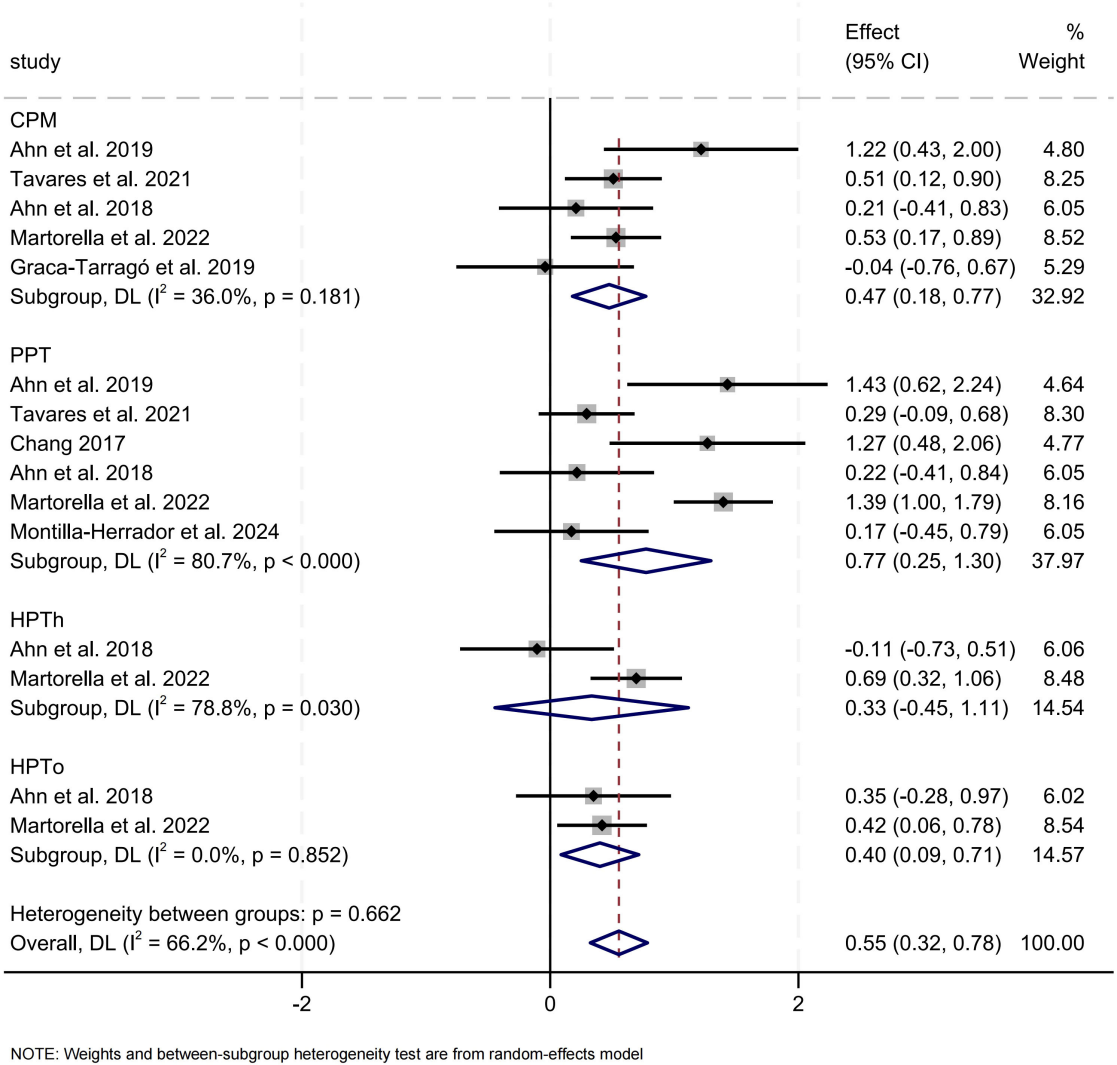
Pooled SMD for change in pain threshold.

Subgroup analyses further revealed that tDCS exerted a significant beneficial effect on CPM (SMD = 0.47, 95% CI: 0.18 to 0.77, *P* < 0.01), HPTo (SMD = 0.40, 95% CI: 0.09 to 0.71, *P* = 0.01) and PPT (SMD = 0.77, 95% CI: 0.25 to 1.30, *P* < 0.01), while no statistically significant difference was observed in HPTh (SMD = 0.33, 95% CI: -0.45 to 1.11, *P* = 0.40). No significant heterogeneity was found among subgroups (*P* = 0.65) (**Figure 2**).

### 3.4 Pain intensity

**Figure 3:**
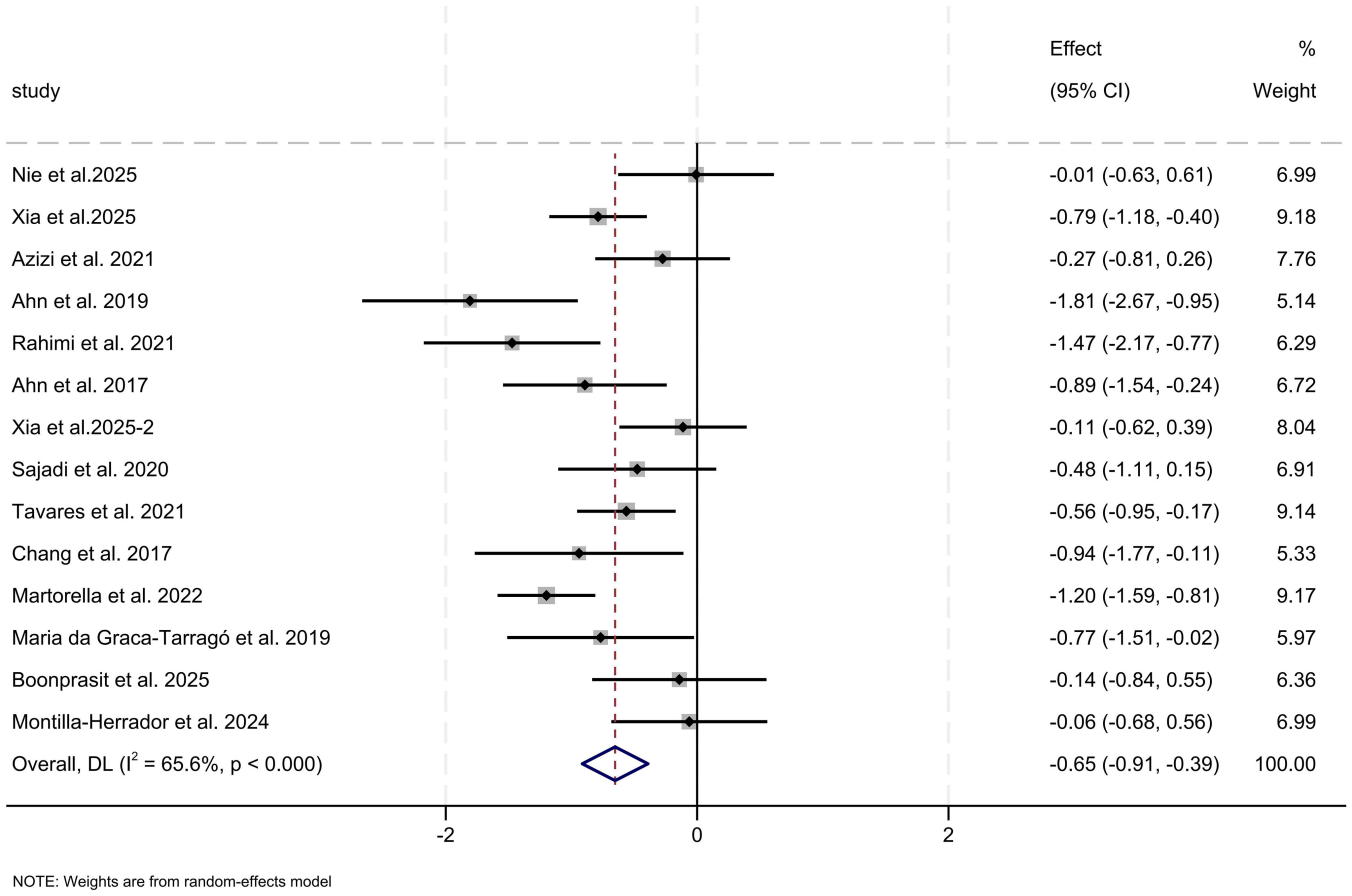
Pooled SMD for change in pain intensity.

A total of 14 studies were included for the analysis of pain intensity, with lower SMD values indicating alleviated clinical pain. The pooled results of the random-effects model showed that tDCS intervention significantly reduced pain scores (VAS/NRS) compared with the control group (SMD = -0.65, 95% CI: -0.91 to -0.39, *P* < 0.01), with moderate between-study heterogeneity (I² = 67.3%) (**Figure 3**). Egger’s test and funnel plot suggested no significant publication bias (*P* = 0.478) (**Supplementary Figure 3**). Leave-one-out sensitivity analysis confirmed the robustness of the pooled effect size (**Supplementary Figure 4**).

### 3.5 Physical function

**Figure 4:**
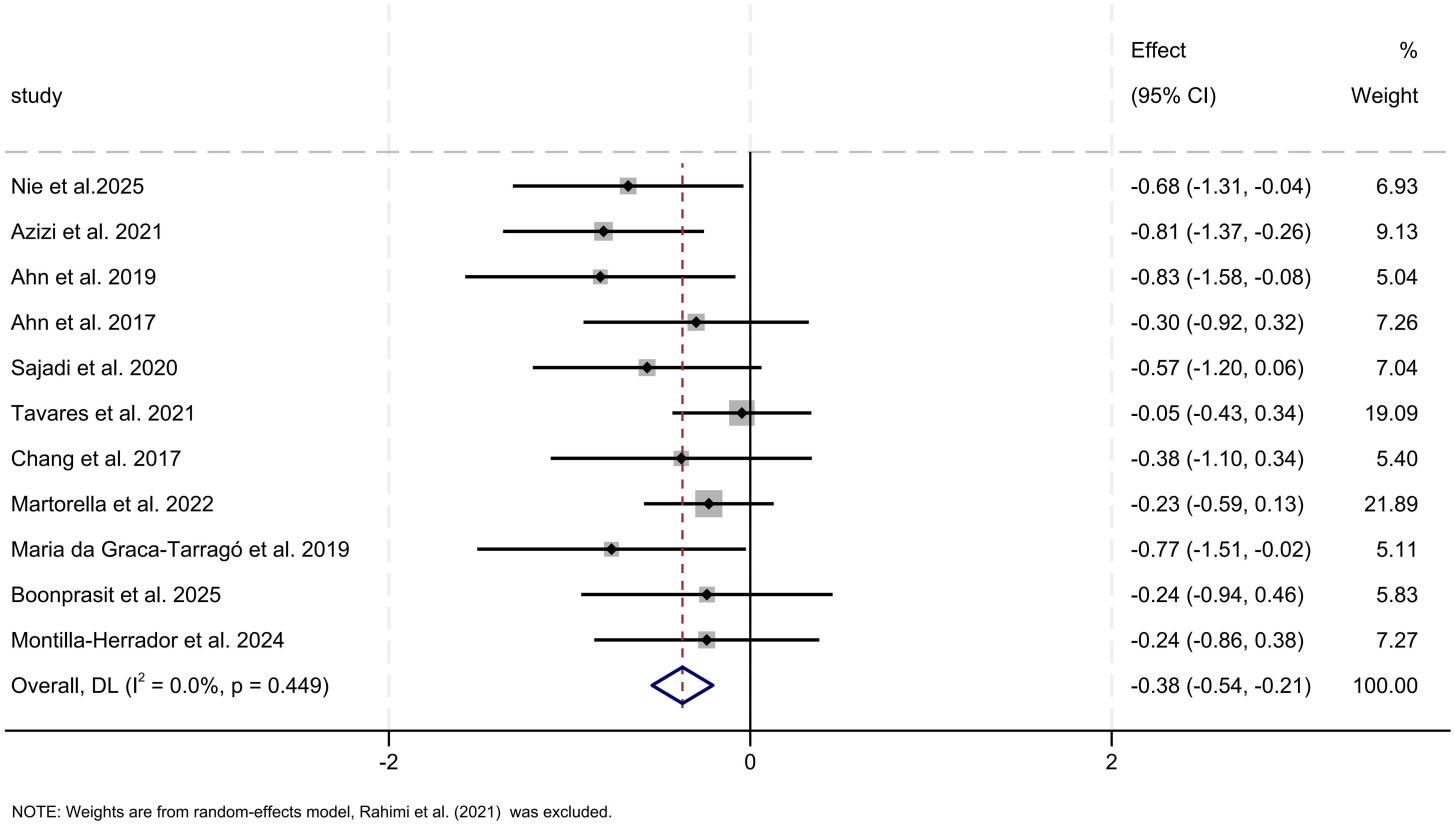
Pooled SMD for change in physical function.

A total of 11 studies were included for the analysis of physical function, with lower SMD values indicating improved knee function (WOMAC/KOOS). The pooled results of the random-effects model showed high between-study heterogeneity initially (I² = 95.29%), and leave-one-out sensitivity analysis indicated that removing the study by Rahimi et al. (2021) markedly reduced heterogeneity (I² = 0.00%) (**Figure 4**). After heterogeneity adjustment, tDCS intervention significantly improved knee physical function compared with the control group (SMD = -0.38, 95% CI: -0.54 to -0.21, *P* < 0.01). Egger’s test and funnel plot suggested no significant publication bias (*P* = 0.054) (**Supplementary Figure 5**). Leave-one-out sensitivity analysis confirmed the robustness of the pooled effect size (**Supplementary Figure 6**).

### 3.6 Walking capacity

**Figure 5:**
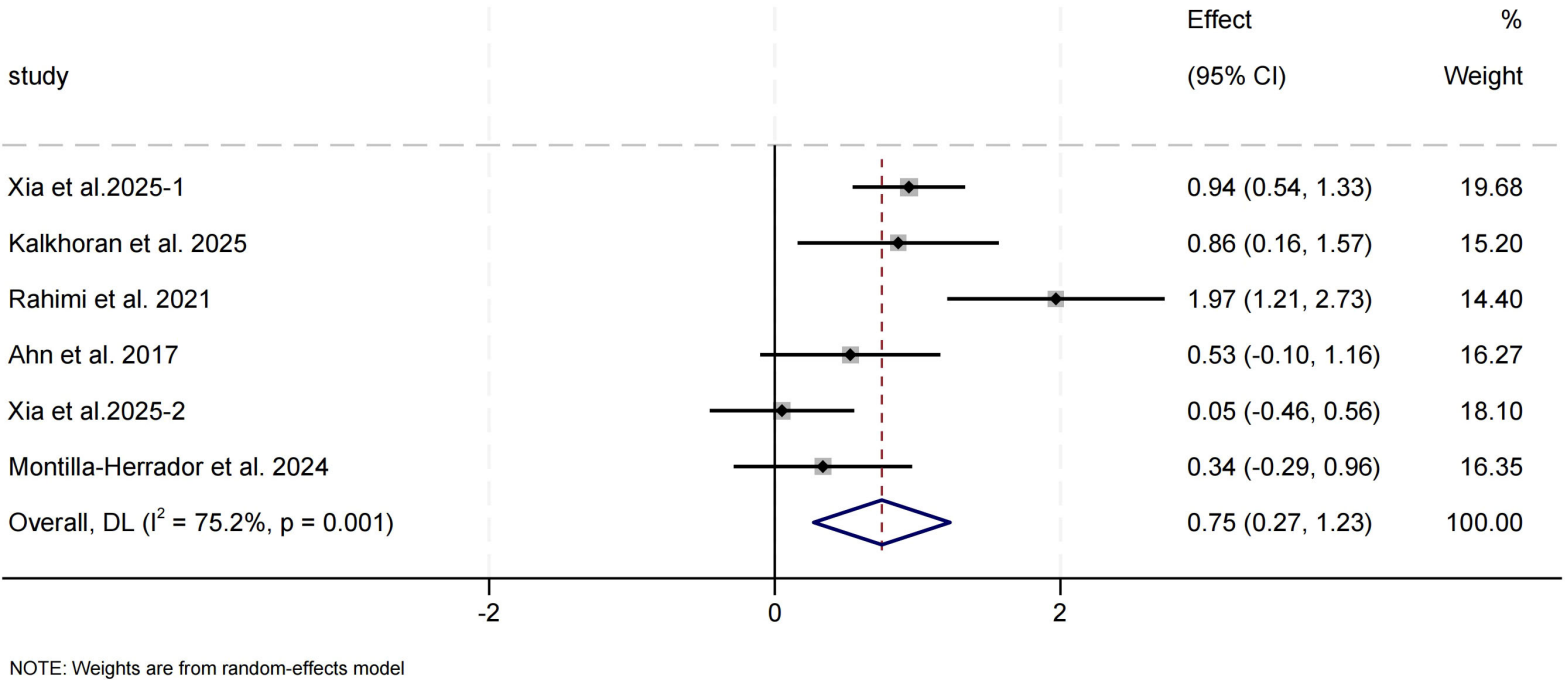
Pooled SMD for change in walking capacity.

A total of 6 studies were included for the analysis of walking capacity, with higher SMD values indicating improved walking ability (6MWT/10MWT). The pooled results of the random-effects model showed that tDCS intervention significantly improved walking capacity compared with the control group (SMD = 0.75, 95% CI: 0.27 to 1.23, *P* < 0.01), with high between-study heterogeneity (I² = 75.2%) (**Figure 5**). Egger’s test and funnel plot suggested no significant publication bias (*P* = 0.346) (**Supplementary Figure 7**). Leave-one-out sensitivity analysis confirmed the robustness of the pooled effect size (**Supplementary Figure 8**).

### 3.7 Risk of bias

**Figure 6:**
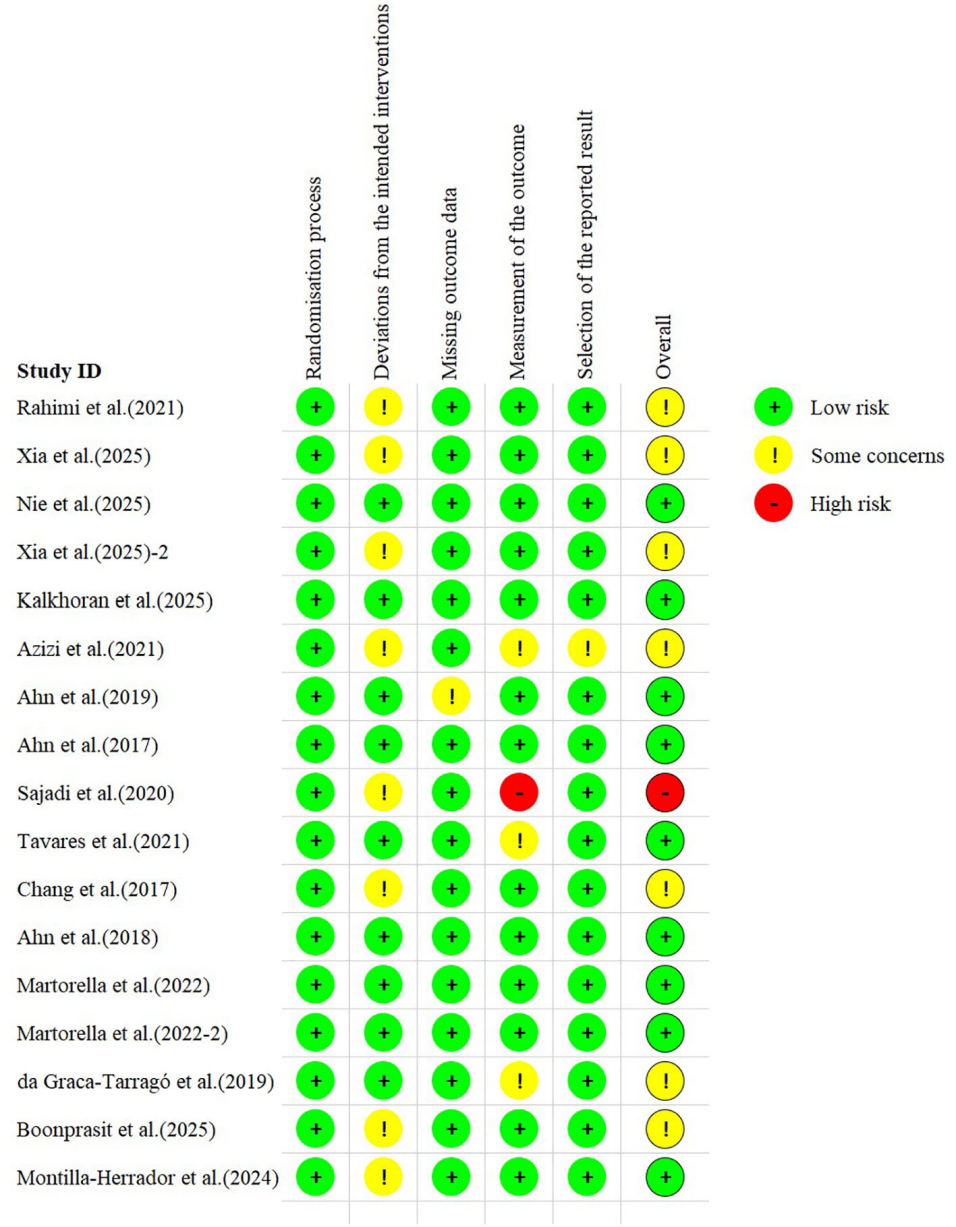
Risk of bias summary.

The RoB 2.0 assessment revealed varying levels of bias among the included studies. Specifically, 9 studies (52.9%) were judged to be at low risk of bias, 7 studies (41.2%) raised some concerns, and 1 study (5.9%) was deemed to be at high risk of bias. All 17 included studies (100%) were rated as low risk in the randomization process domain. The “deviations from intended interventions” domain was identified as the primary source of uncertainty, with 8 studies (47.1%) raising some concerns. Most studies had low risk in the missing outcome data domain, with 16 studies (94.1%) classified as low risk. With respect to the outcome measurement domain, 13 studies (76.5%) were rated as low risk, 3 studies (17.6%) raised some concerns, and 1 study (5.9%) was classified as high risk. Sensitivity analyses excluding high-risk studies showed that the direction of the pooled effects was generally unchanged. However, outcomes with high heterogeneity, especially physical function and walking capacity, should be interpreted cautiously because studies with some concerns or high risk of bias may affect the precision of the estimates. **Figure 6** displays the results of the risk of bias assessment.

### 3.8 Adverse events

Ten studies reported adverse events, while seven documented no adverse reactions or favorable tolerability. No severe intervention-related adverse events were identified. Most adverse events were mild and transient, consisting mainly of scalp tingling, itching, pain, local redness, or irritation. A small number of patients reported headache, dizziness, or fatigue. Minor bleeding after needle removal was observed in trials combined with acupuncture. Only 10 patients discontinued due to adverse events (scalp discomfort or mild headache) in two studies.

## 4 Discussion

Previous studies have mainly focused on pain perception and joint function, with relatively insufficient attention paid to pain threshold (Wu et al., 2024). The present study demonstrated that tDCS significantly improved pain perception in patients with KOA. Consistent with these results, the pooled effect showed that tDCS significantly relieved pain and improved knee function in KOA patients, with favorable clinical safety. To the best of our knowledge, this is the first systematic review and meta-analysis investigating the effects of tDCS on KOA with pain threshold as the primary outcome.

### 4.1 Pain threshold

The present study found that tDCS was significantly superior to control interventions in increasing pain threshold. Subgroup analyses revealed that tDCS significantly improved CPM, PPT, and HPTo, whereas no significant effect was observed for HPTh. Pain threshold, also known as pain sensitivity, is considered an important indicator of central sensitization (Nijs et al., 2021). These findings suggest that the therapeutic effect of tDCS in KOA may mainly target pain modulation and central sensitization, which is consistent with the central sensitization theory of KOA (Fingleton et al., 2015; Arant et al., 2022). Current evidence on central sensitization in KOA indicates that persistent tissue injury and joint inflammation can induce hyperexcitability of pain pathways in the central nervous system, thereby triggering central sensitization (Nijs et al., 2021; Xiong et al., 2024). Correspondingly, Lan et al. (2020) using fMRI reported a global reorganization of nodal centrality in brain networks at rest in KOA patients. Previous randomized controlled trials have shown that 15 sessions of tDCS over the primary motor cortex significantly reduced pain scores and improved conditioned pain modulation in elderly KOA patients (Tavares et al., 2021). Nie et al. (2025) also found that tDCS combined with acupuncture was more effective than acupuncture alone in reducing the connectivity strength of pain−related brain networks. The observed improvement in pain threshold in the present study further supports that tDCS can modulate central sensitization in KOA patients (Salazar-Méndez et al., 2023). Meanwhile, the reduction in pain scores was consistent with the increase in pain threshold, providing mutually supportive evidence for clinical benefits and neurobiological changes.

### 4.2 Improvement in clinical function

Clinical functional improvement in KOA patients generally includes pain relief and recovery of knee joint function (Martorella et al., 2022b). The present study supports that tDCS can alleviate pain perception and promote functional recovery in KOA patients by regulating central pain processing and neuroplasticity. A study by Martorella et al. (2022b) showed that self−administered tDCS at home for 3 weeks significantly reduced pain intensity in elderly KOA patients, suggesting that tDCS is feasible and safe for pain relief.

However, Wu et al. (2024) reported that although tDCS effectively relieved pain, tDCS alone did not significantly improve physical function (e.g., WOMAC, KOOS scores) in KOA patients, indicating inconsistent effects of tDCS monotherapy on functional recovery. Regarding functional rehabilitation, (Montilla-Herrador et al., 2024). added tDCS or transcutaneous electrical nerve stimulation (TENS) to an educational and active exercise program. The results showed that the basic program itself improved pain and function, but the additional effect of tDCS was not significantly superior to the basic rehabilitation intervention. A plausible explanation for this lack of incremental benefit is that standard rehabilitation may have reached a ceiling effect, thereby obscuring the true efficacy of adjunct tDCS.

Sajadi et al. (2020) examined the combined effects of tDCS and TENS and found that the combined regimen further improved pain and function in KOA patients, but no statistically significant difference was observed between groups. These discrepancies across studies contributed to the high heterogeneity in the pooled results of this meta−analysis.

### 4.3 Heterogeneity and effect modifiers

The present study and existing systematic reviews have indicated that tDCS exhibits a relatively consistent effect on pain relief in patients with KOA. However, significant heterogeneity is observed in outcome measures related to function and walking capacity, such as WOMAC, KOOS, and 6MWT. First, the functional assessment tools are inconsistent. Different studies adopt WOMAC, KOOS, or various walking/functional tests, which are not completely equivalent in their clinical domains and sensitivity. Differences in assessment tools (6MWT vs. 10MWT) and baseline pain severity may contribute to the observed heterogeneity. This makes it difficult to directly compare functional improvements of the same intervention across studies, thereby increasing the variability in effect estimates (Emery et al., 2019). Second, there are substantial differences in study design. Some trials combine tDCS with physical training, TENS, or other interventions, whereas others use tDCS alone. The inconsistency in intervention protocols and tDCS parameters contributes to methodological heterogeneity (O'Connell et al., 2018; Gonzalez et al., 2021). M1-targeted tDCS has been associated with pain modulation and descending inhibitory function in KOA, whereas DLPFC stimulation may involve cognitive-affective pain regulation (Seminowicz and Moayedi, 2017). Third, differences in patient phenotypes may modify the treatment effect. For instance, the degree of central pain sensitization and psychological characteristics can influence individual responses to tDCS, thus introducing variability in efficacy (Yang et al., 2024). In conclusion, although tDCS is overall effective for patients with KOA, its effect size and reproducibility are highly dependent on the choice of assessment tools, intervention design, and baseline patient characteristics.

### 4.4 Implications for clinical research

Future trials should use a uniform framework to record and report adverse events. This will improve the comparability and reliability of safety evidence. Current evidence suggests that tDCS can be a complementary central neuromodulatory approach in the non-surgical, multimodal management of knee osteoarthritis, particularly for patients with central sensitization. To advance this field, future clinical studies should focus on enrolling participants based on mechanistic phenotypes. Standardization of stimulation parameters is essential, along with uniform measurement protocols for QST and CPM to ensure reproducibility and comparability across studies. Furthermore, mediation analysis frameworks should be adopted to enable the development of mechanism-driven therapeutic strategies. The low dropout rate due to adverse events supports that tDCS is safe and repeatable. It can be integrated into routine rehabilitation.

### 4.5 Limitations

The conclusions of this study must be viewed in light of several limitations. First, considerable variability in participant characteristics, tDCS parameters (current intensity, duration, number of sessions), and combined intervention protocols across trials resulted in significant heterogeneity, limiting the robustness and generalizability of the effect sizes. Second, the lack of standardization in measurement tools and protocols for mechanistic outcomes may affect comparability between studies. Third, most studies had small sample sizes, short follow-up periods, and varying risks of bias, with inconsistent reporting of adverse events, leading to insufficient evidence on long-term efficacy and safety.

## 5 Conclusion

Current evidence from randomized controlled trials suggests that tDCS may improve pain threshold and reduce pain intensity in patients with KOA. The effects on physical function and walking capacity appear favorable but remain less certain because of substantial heterogeneity and potential risk-of-bias effects. Overall, tDCS may serve as a promising central neuromodulatory adjunct for KOA pain management, particularly in patients with altered pain processing. Nevertheless, substantial heterogeneity across studies limits the certainty of these findings, and future high-quality RCTs using mechanistic stratification and standardized stimulation and assessment protocols are warranted.

## Data Availability

The original contributions presented in the study are included in the article/**Supplementary Material**. Further inquiries can be directed to the corresponding author.

## References

[B29] Ahn H. Suchting R. Woods A. J. Miao H. Green C. Cho R.Y. . (2018). Bayesian analysis of the effect of transcranial direct current stimulation on experimental pain sensitivity in older adults with knee osteoarthritis: randomized sham-controlled pilot clinical study. J. Pain Res. 11, 2071–2082. doi: 10.2147/jpr.S173080 30310309 PMC6166765

[B24] Ahn H. Woods A. J. Kunik M. E. Bhattacharjee A. Chen Z. Choi E. . (2017). Efficacy of transcranial direct current stimulation over primary motor cortex (anode) and contralateral supraorbital area (cathode) on clinical pain severity and mobility performance in persons with knee osteoarthritis: an experimenter- and participant-blinded, randomized, sham-controlled pilot clinical study. Brain Stimulation 10, 902–909. doi: 10.1016/j.brs.2017.05.007 28566193 PMC5568498

[B22] Ahn H. Zhong C. Miao H. Chaoul A. Park L. Yen I. H. . (2019). Efficacy of combining home-based transcranial direct current stimulation with mindfulness-based meditation for pain in older adults with knee osteoarthritis: a randomized controlled pilot study. J. Clin. Neurosci. Off. J. Neurosurgical Soc. Australasia 70, 140–145. doi: 10.1016/j.jocn.2019.08.047 31421990

[B6] Aoyagi K. Liew J. W. Farrar J. T. Wang N. Carlesso L. Kumar D. . (2022). Does weight-bearing versus non-weight-bearing pain reflect different pain mechanisms in knee osteoarthritis?: the Multicenter Osteoarthritis Study (MOST). Osteoarthritis Cartilage 30, 545–550. doi: 10.1016/j.joca.2021.10.014 34801670 PMC8940656

[B8] Arant K. R. Katz J. N. Neogi T. (2022). Quantitative sensory testing: identifying pain characteristics in patients with osteoarthritis. Osteoarthritis Cartilage 30, 17–31. doi: 10.1016/j.joca.2021.09.011 34597800 PMC8712382

[B21] Azizi S. Rezasoltani Z. Najafi S. Mohebi B. Tabatabaee S. M. Dadarkhah A. (2021). Transcranial direct current stimulation for knee osteoarthritis: a single-blind randomized sham-controlled trial. Neurophysiologie Clinique = Clin. Neurophysiol. 51, 329–338. doi: 10.1016/j.neucli.2020.12.002 33323306

[B4] Bannuru R. R. Osani M. C. Vaysbrot E. E. Arden N. K. Bennell K. Bierma-Zeinstra S. M. A. . (2019). OARSI guidelines for the non-surgical management of knee, hip, and polyarticular osteoarthritis. Osteoarthritis Cartilage 27, 1578–1589. doi: 10.1016/j.joca.2019.06.011 31278997

[B33] Boonprasit K. Stonsaovapak C. Piravej K. (2025). Effect of transcranial direct current stimulation at the left dorsolateral prefrontal cortex combined with quadriceps strengthening exercise in chronic knee osteoarthritis: a double-blind, randomized, sham-controlled trial. Am. J. Phys. Med. Rehabil. 105 (3), 200–207. doi: 10.1097/phm.0000000000002852 40907993

[B15] Brignardello-Petersen R. Bonner A. Alexander P. E. Siemieniuk R.A. Furukawa T.A. Rochwerg B. . (2018). Advances in the GRADE approach to rate the certainty in estimates from a network meta-analysis. J. Clin. Epidemiol. 93, 36–44. doi: 10.1016/j.jclinepi.2017.10.005 29051107

[B28] Chang W. J. Bennell K. L. Hodges P. W. Hinman R.S. Young C.L. Buscemi V. . (2017). Addition of transcranial direct current stimulation to quadriceps strengthening exercise in knee osteoarthritis: a pilot randomised controlled trial. PloS One 12, e0180328. doi: 10.1371/journal.pone.0180328 28665989 PMC5493377

[B2] Cui A. Li H. Wang D. Zhong J. Chen Y. Lu H. (2020). Global, regional prevalence, incidence and risk factors of knee osteoarthritis in population-based studies. EClinicalMedicine 29-30, 100587. doi: 10.1016/j.eclinm.2020.100587 34505846 PMC7704420

[B32] da Graca-Tarragó M. Lech M. Angoleri L. D. M. Santos D. S. Deitos A. Brietzke A. P. . (2019). Intramuscular electrical stimulus potentiates motor cortex modulation effects on pain and descending inhibitory systems in knee osteoarthritis: a randomized, factorial, sham-controlled study. J. Pain Res. 12, 209–221. doi: 10.2147/jpr.S181019 30655690 PMC6322702

[B11] Dehghani A. Gantz D. M. Murphy E. K. Nitsche M. A. Halter R. J. Wager T. D. (2025). Transcranial direct current stimulation of primary motor cortex reduces thermal pain. Pain. 167 (4), 880–892. doi: 10.1097/j.pain.0000000000003851 41380142 PMC12994524

[B3] Duong V. Oo W. M. Ding C. Culvenor A. G. Hunter D. J. (2023). Evaluation and treatment of knee pain: a review. Jama 330, 1568–1580. doi: 10.1001/jama.2023.19675 37874571

[B41] Emery C. A. Whittaker J. L. Mahmoudian A. Lohmander L.S. Roos E.M. Bennell K.L. . (2019). Establishing outcome measures in early knee osteoarthritis. Nat. Rev. Rheumatol. 15, 438–448. doi: 10.1038/s41584-019-0237-3 31201386

[B37] Fingleton C. Smart K. Moloney N. Fullen B. M. Doody C. (2015). Pain sensitization in people with knee osteoarthritis: a systematic review and meta-analysis. Osteoarthritis Cartilage 23, 1043–1056. doi: 10.1016/j.joca.2015.02.163 25749012

[B1] Gelber A. C. (2024). Knee osteoarthritis. Ann. Internal Med. 177, Itc129–itc144. doi: 10.7326/annals-24-01249 39250809

[B42] Gonzalez P. C. Fong K. N. K. Brown T. (2021). Transcranial direct current stimulation as an adjunct to cognitive training for older adults with mild cognitive impairment: a randomized controlled trial. Ann. Phys. Rehabil. Med. 64, 101536. doi: 10.1016/j.rehab.2021.101536 33957292

[B17] Higgins J. P. Thompson S. G. Deeks J. J. Altman D. G. (2003). Measuring inconsistency in meta-analyses. BMJ (Clinical Res. Ed) 327, 557–560. doi: 10.1136/bmj.327.7414.557 12958120 PMC192859

[B20] Kalkhoran Z. B. Khanmohammadi R. (2025). The effects of aquatic therapy combined with transcranial direct current stimulation (tDCS) on proprioception and gait speed in older adults with knee osteoarthritis: an eight-week randomized sham-controlled trial. BMC Geriatrics 25, 676. doi: 10.1186/s12877-025-06253-5 40898048 PMC12403502

[B39] Lan F. Lin G. Cao G. Li Z. Ma D. Liu F. . (2020). Altered intrinsic brain activity and functional connectivity before and after knee arthroplasty in the elderly: a resting-state fMRI study. Front. Neurol. 11, 556028. doi: 10.3389/fneur.2020.556028 33133006 PMC7550714

[B13] Lawford B. J. Bennell K. L. Haber T. Hall M. Hinman R.S. Recenti F. . (2024). Osteoarthritis year in review 2024: rehabilitation and outcomes. Osteoarthritis Cartilage 32, 1405–1412. doi: 10.1016/j.joca.2024.08.001 39116992

[B14] Liberati A. Altman D. G. Tetzlaff J. Mulrow C. Gøtzsche P. C. Ioannidis J. P. . (2009). The PRISMA statement for reporting systematic reviews and meta-analyses of studies that evaluate health care interventions: explanation and elaboration. PloS Med. 6, e1000100. doi: 10.1371/journal.pmed.1000100 19621070 PMC2707010

[B10] Lloyd D. M. Wittkopf P. G. Arendsen L. J. Jones A. K. P. (2020). Is transcranial direct current stimulation (tDCS) effective for the treatment of pain in fibromyalgia? A systematic review and meta-analysis. J. Pain 21, 1085–1100. doi: 10.1016/j.jpain.2020.01.003 31982685

[B30] Martorella G. Mathis K. Miao H. Wang D. Park L. Ahn H. (2022a). Efficacy of home-based transcranial direct current stimulation on experimental pain sensitivity in older adults with knee osteoarthritis: a randomized, sham-controlled clinical trial. J. Clin. Med. 11 (17), 5209. doi: 10.3390/jcm11175209 36079139 PMC9457351

[B31] Martorella G. Mathis K. Miao H. Wang D. Park L. Ahn H. (2022b). Self-administered transcranial direct current stimulation for pain in older adults with knee osteoarthritis: a randomized controlled study. Brain Stimulation 15, 902–909. doi: 10.1016/j.brs.2022.06.003 35690388 PMC9387776

[B34] Montilla-Herrador J. Lozano-Meca J. Lozano-Guadalajara J. V. Gacto-Sánchez M. (2024). The efficacy of the addition of tDCS and TENS to an education and exercise program in subjects with knee osteoarthritis: a randomized controlled trial. Biomedicines 12 (6), 1186. doi: 10.3390/biomedicines12061186 38927392 PMC11200463

[B5] Moseng T. Vliet Vlieland T. P. M. Battista S. Battista S. Beckwée D. Boyadzhieva V. Conaghan P. G. . (2024). EULAR recommendations for the non-pharmacological core management of hip and knee osteoarthritis: 2023 update. Ann. Rheumatic Dis. 83, 730–740. doi: 10.1136/ard-2023-225041 38212040 PMC11103326

[B18] Nie Q. He F. Dong L. Lin X. Lin J. Wang Y. . (2025). Effects of transcranial direct current stimulation combined with acupuncture therapy on brain network functional connectivity in patients with knee osteoarthritis: a single-center randomized controlled trial. J. NeuroEng. Rehabil. 22, 160. doi: 10.1186/s12984-025-01692-y 40660313 PMC12257676

[B36] Nijs J. George S. Z. Clauw D. J. Fernández-de-Las-Peñas C. Kosek E. Ickmans K. . (2021). Central sensitisation in chronic pain conditions: latest discoveries and their potential for precision medicine. Lancet Rheumatol. 3, e383–e392. doi: 10.1016/s2665-9913(21)00032-1 38279393

[B43] O'Connell N. E. Marston L. Spencer S. DeSouza L. H. Wand B. M. (2018). Non-invasive brain stimulation techniques for chronic pain. Cochrane Database Systematic Rev. 4, Cd008208. doi: 10.1002/14651858.CD008208.pub5 29652088 PMC6494527

[B9] Petersen K. K. Kilic K. Hertel E. Sejersgaard-Jacobsen T. H. Jørgensen M. K. Troelsen A. . (2023). Quantitative sensory testing as an assessment tool to predict the response to standard pain treatment in knee osteoarthritis: a systematic review and meta-analysis. Pain Rep. 8, e1079. doi: 10.1097/pr9.0000000000001079 38699564 PMC11065125

[B23] Rahimi F. Nejati V. Nassadj G. Ziaei B. Mohammadi H. K. (2021). The effect of transcranial direct stimulation as an add-on treatment to conventional physical therapy on pain intensity and functional ability in individuals with knee osteoarthritis: a randomized controlled trial. Neurophysiologie Clinique = Clin. Neurophysiol. 51, 507–516. doi: 10.1016/j.neucli.2021.06.002 34518098

[B12] Rodríguez-Lagos L. Fernández-Carnero J. Laguarta-Val S. Serrano-García B. Martín-Vera D. Runge N. . (2025). Conditioned pain modulation and temporal summation in patients with knee osteoarthritis: a systematic review and meta-analysis. J. Pain 33, 105464. doi: 10.1016/j.jpain.2025.105464 40517980

[B26] Sajadi S. Karimi M. Forogh B. Raissi G. R. Zarnegar F. Ahadi T. (2020). Randomized clinical trial comparing of transcranial direct current stimulation (tDCS) and transcutaneous electrical nerve stimulation (TENS) in knee osteoarthritis. Neurophysiologie Clinique = Clin. Neurophysiol. 50, 367–374. doi: 10.1016/j.neucli.2020.08.005 32912627

[B40] Salazar-Méndez J. Cuyul-Vásquez I. Viscay-Sanhueza N. Morales-Verdugo J. Mendez-Rebolledo G. Ponce-Fuentes F. . (2023). Structural and functional brain changes in people with knee osteoarthritis: a scoping review. PeerJ 11, e16003. doi: 10.7717/peerj.16003 37701842 PMC10493091

[B7] Satake Y. Izumi M. Aso K. Igarashi Y. Sasaki N. Ikeuchi M. (2021). Comparison of predisposing factors between pain on walking and pain at rest in patients with knee osteoarthritis. J. Pain Res. 14, 1113–1118. doi: 10.2147/jpr.S298100 33907458 PMC8068486

[B44] Seminowicz D. A. Moayedi M. (2017). The dorsolateral prefrontal cortex in acute and chronic pain. J. Pain 18, 1027–1035. doi: 10.1016/j.jpain.2017.03.008 28400293 PMC5581265

[B16] Sterne J. A. C. Savović J. Page M. J. Elbers R.G. Blencowe N.S. Boutron I. . (2019). RoB 2: a revised tool for assessing risk of bias in randomised trials. BMJ (Clinical Res. Ed) 366, l4898. doi: 10.1136/bmj.l4898 31462531

[B27] Tavares D. R. B. Okazaki J. E. F. Santana M. V. A. Pinto A. Tutiya K. K. Gazoni F. M. . (2021). Motor cortex transcranial direct current stimulation effects on knee osteoarthritis pain in elderly subjects with dysfunctional descending pain inhibitory system: a randomized controlled trial. Brain Stimulation 14, 477–487. doi: 10.1016/j.brs.2021.02.018 33684598

[B35] Wu Y. L. Luo Y. Yang J. M. Wu Y. Q. Zhu Q. Li Y. . (2024). Effects of transcranial direct current stimulation on pain and physical function in patients with knee osteoarthritis: a systematic review and meta-analysis. BMC Musculoskeletal Disord. 25, 703. doi: 10.1186/s12891-024-07805-3 39227806 PMC11370230

[B19] Xia C. Y. Niu H. Z. Zhang X. W. Cai S. Y. Fan Y. J. Xie T. P. . (2025a). Transcranial direct current stimulation enhances the efficacy of wearable transcutaneous electrical nerve stimulation for mild knee osteoarthritis in the middle-aged person: a randomized controlled trial. BMC Musculoskeletal Disord. 26, 684. doi: 10.1186/s12891-025-08924-1 40665284 PMC12261559

[B25] Xia C. Y. Tian H. F. Ren X. Y. Xiao Z. H. Chen H. A. Yin Y. J. . (2025b). Investigate the efficacy of dual-target electrical stimulation in the treatment of knee osteoarthritis after stroke and its effect on cerebral cortical activity: a randomized controlled trial. Neural Plast. 2025, 2886215. doi: 10.1155/np/2886215 40809334 PMC12350009

[B38] Xiong H. Y. Hendrix J. Schabrun S. Wyns A. Campenhout J. V. Nijs J. . (2024). The role of the brain-derived neurotrophic factor in chronic pain: links to central sensitization and neuroinflammation. Biomolecules 14 (1), 71. doi: 10.3390/biom14010071 38254671 PMC10813479

[B45] Yang J. M. Li C. C. Wang Y. Li J. Y. Xu J. M. Liang M. G. . (2024). Transcranial direct current stimulation for knee osteoarthritis: a systematic review and meta-analysis of randomized controlled trials. Arthritis Care Res. 76, 376–384. doi: 10.1002/acr.25249 37779486

