# Supplementary material for: Transcranial direct current stimulation for pain threshold in knee osteoarthritis: a mechanism-oriented systematic review and meta-analysis of randomized controlled trials
Source: Front Physiol. 2026 Jul 14;17:1890510. doi: 10.3389/fphys.2026.1890510 (PMC13407280; doi:10.3389/fphys.2026.1890510)
Supplement: Supplementary file 1 [file SupplementaryFile1.docx]

**Supplemental materials content**

**Part A**

Search strategy

**Part B**

**Figure S1.** Funnel plot of the pain threshold.

**Figure S2.** Sensitivity analysis of the pain threshold.

**Figure S3.** Funnel plot of the pain intensity.

**Figure S4.** Sensitivity analysis of the pain intensity.

**Figure S5.** Funnel plot of the physical function.

**Figure S6.** Sensitivity analysis of the physical function.

**Figure S7.** Funnel plot of the walk capacity.

**Figure S8.** Sensitivity analysis of the walk capacity.

**Part A**

**The search results of each database are as follows: 57 results in PubMed; 51 results in Web of Science; 90 results in Embase; 101 results in Cochrane.**

**PubMed**

#1 transcranial direct current stimulation[MeSH Terms] AND transcranial direct current stimulation[Title/Abstract] OR transcranial direct-current stimulation[Title/Abstract] OR tDCS[Title/Abstract] OR anodal tDCS[Title/Abstract] OR cathodal tDCS[Title/Abstract]

#2 Knee Osteoarthritides[Title/Abstract] OR Knee Osteoarthritis[Title/Abstract] OR Osteoarthritis of Knee[Title/Abstract] OR Osteoarthritis of the Knee [Title/Abstract] AND Osteoarthritis, Knee*[Title/Abstract] OR Knee* Osteoarthritis [Title/Abstract] OR KOA[Title/Abstract] OR Osteoarthritis of Knee*[Title/Abstract] OR Degenerative Joint Disease Knee*[Title/Abstract] OR Ostarthritis, Knee*[Title/Abstract] OR Degenerative Arthritis, Knee* [Title/Abstract] OR Degenerative Arthritis, Knee*[Title/Abstract]

#3: #1 AND #2: (transcranial direct current stimulation[MeSH Terms] AND transcranial direct current stimulation[Title/Abstract] OR transcranial direct-current stimulation[Title/Abstract] OR tDCS[Title/Abstract] OR anodal tDCS[Title/Abstract] OR cathodal tDCS[Title/Abstract]) AND (Knee Osteoarthritides[Title/Abstract] OR Knee Osteoarthritis[Title/Abstract] OR Osteoarthritis of Knee[Title/Abstract] OR Osteoarthritis of the Knee [Title/Abstract] AND Osteoarthritis, Knee*[Title/Abstract] OR Knee* Osteoarthritis [Title/Abstract] OR KOA[Title/Abstract] OR Osteoarthritis of Knee*[Title/Abstract] OR Degenerative Joint Disease Knee*[Title/Abstract] OR Ostarthritis, Knee*[Title/Abstract] OR Degenerative Arthritis, Knee* [Title/Abstract] OR Degenerative Arthritis, Knee*[Title/Abstract])

We have found **57** articles that used the keywords above.

**web of sciense**

#1: TS=(transcranial direct current stimulation) OR AB=(transcranial direct-current stimulation) OR AB=(tDCS) OR AB=(anodal tDCS) OR AB=(cathodal tDCS) OR AB=(transcranial direct-current stimulation) OR AB=(tDCS) OR AB=(anodal transcranial direct current stimulation) OR AB=(anodal tDCS) OR AB=(cathodal transcranial direct current stimulation) OR AB=(cathodal tDCS) OR AB=(transcranial alternating current stimulation)

#2: AB=( Knee Osteoarthritides) OR AB=( Knee Osteoarthritis) OR AB=( Osteoarthritis of Knee) OR AB=( Osteoarthritis of the Knee) OA AB=(Osteoarthritis, Knee*) OR AB=(Knee* Osteoarthritis) OR AB=(KOA) OR AB=(Osteoarthritis of Knee*) OR AB=(Degenerative Joint Disease Knee*) OR AB=( Ostarthritis, Knee*) OR AB=(Degenerative Arthritis, Knee*)

#3: #1 AND #2

We have found **51** articles that used the keywords above.

**Embase**

#1: ' transcranial direct current stimulation ':ab,ti OR ' transcranial direct current stimulation ':ab,ti OR ' transcranial direct-current stimulation ':ab,ti OR ' tDCS ':ab,ti OR ' anodal tDCS ':ab,ti OR ' cathodal tDCS ':ab,ti OR ' transcranial direct-current stimulation ':ab,ti OR ' anodal transcranial direct current stimulation ':ab,ti OR ' transcranial direct-current stimulation ':ab,ti OR 'anodal tDCS ':ab,ti OR ' cathodal transcranial direct current stimulation ':ab,ti OR ' transcranial alternating current stimulation ':ab,ti

#2: ' Knee Osteoarthritides ':ab,ti OR ' Knee Osteoarthritis ':ab,ti OR ' Osteoarthritis of Knee ':ab,ti OR ' Osteoarthritis of the Knee ':ab,ti OR ' Osteoarthritis, Knee* ':ab,ti OR ' Knee* Osteoarthritis ':ab,ti OR ' Osteoarthritis of Knee* ':ab,ti OR ' Degenerative Joint Disease Knee*':ab,ti OR ' Ostarthritis, Knee* ':ab,ti OR ' Degenerative Arthritis, Knee*':ab,ti

#3: #1 AND #2

We have found **90** articles that used the keywords above.

**Cochrane library**

#1: (transcranial direct current stimulation):ti,ab,kw OR (transcranial direct-current stimulation):ti,ab,kw OR (tDCS):ti,ab,kw OR (anodal tDCS):ti,ab,kw OR (cathodal tDCS):ti,ab,kw (anodal transcranial direct current stimulation):ti,ab,kw OR (transcranial direct-current stimulation):ti,ab,kw OR (tDCS):ti,ab,kw OR (transcranial alternating current stimulation):ti,ab,kw

#2: (Knee Osteoarthritides):ti,ab,kw OR (Knee Osteoarthritis):ti,ab,kw OR (Osteoarthritis of Knee):ti,ab,kw OR (Osteoarthritis of the Knee):ti,ab,kw OR (Osteoarthritis, Knee*):ti,ab,kw OR (Knee* Osteoarthritis):ti,ab,kw OR (Osteoarthritis of Knee*):ti,ab,kw OR (Degenerative Joint Disease Knee*):ti,ab,kw OR (Ostarthritis, Knee*):ti,ab,kw OR (Degenerative Arthritis, Knee*):ti,ab,kw

**#1 AND #2**

We have found **101** articles that used the keywords above.

**Part B**


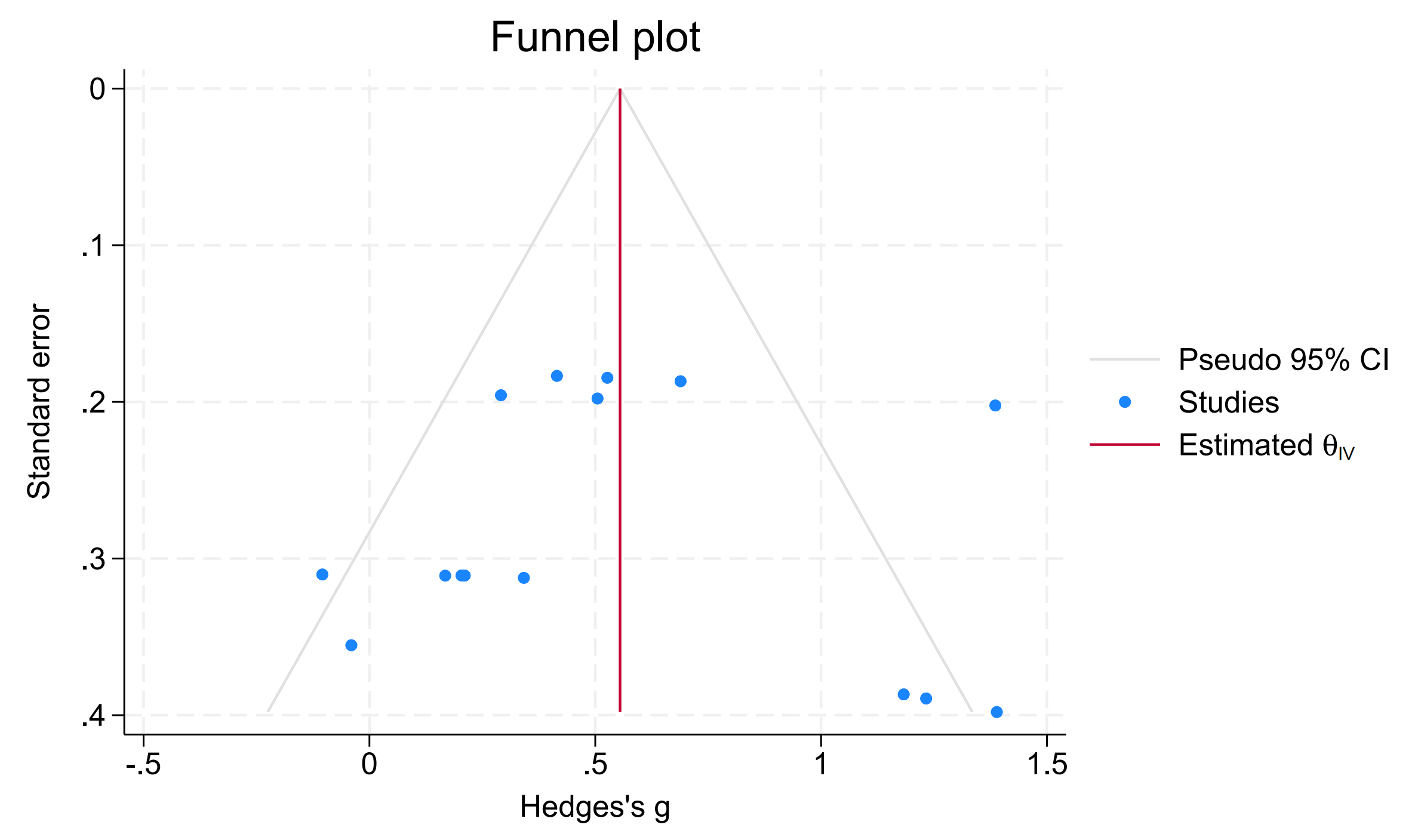


**Figure S1**. Funnel plot of the pain threshold.

**Figure S2**. Sensitivity analysis of the pain threshold.


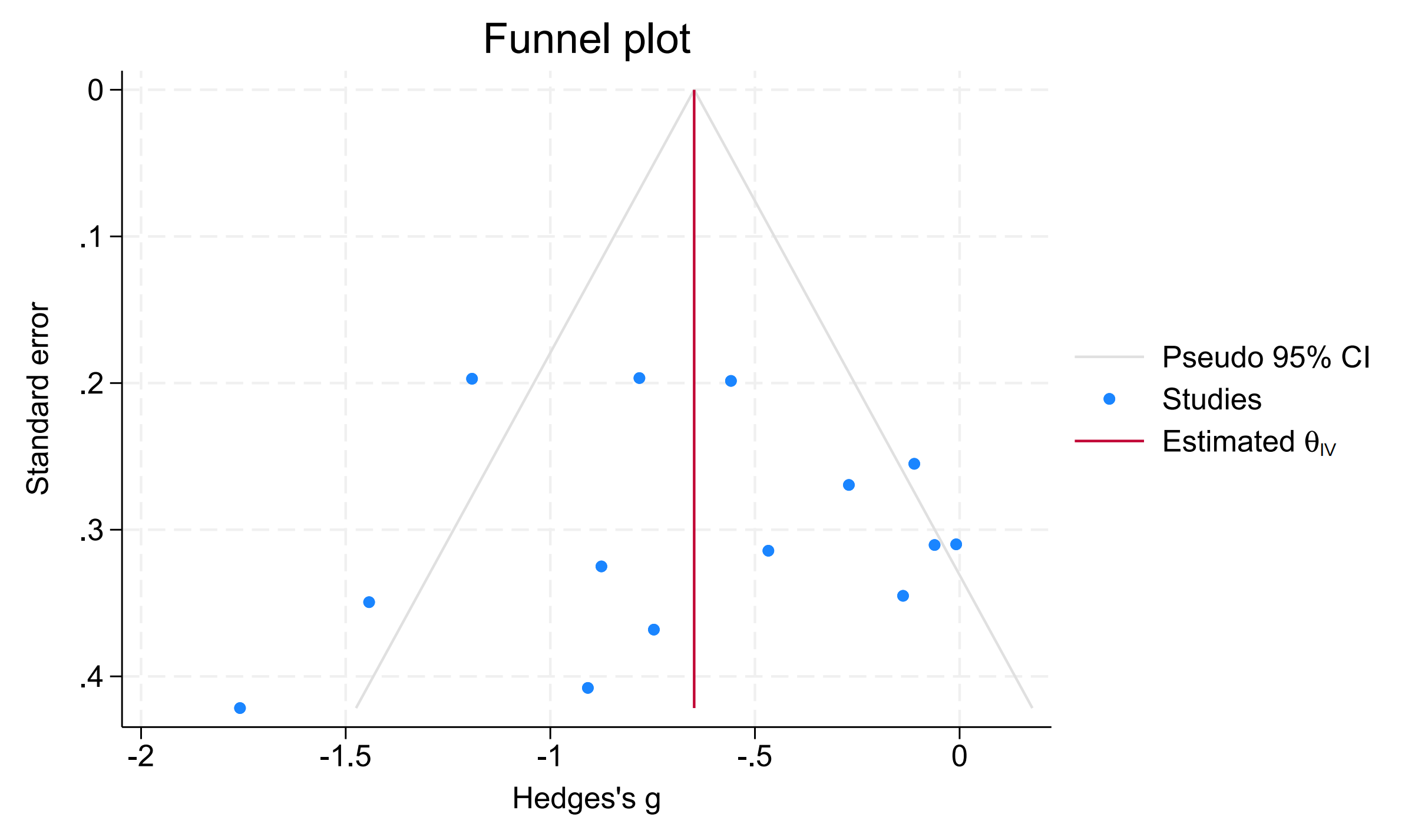


**Figure S3**. Funnel plot of the pain intensity.

**Figure S4**. Sensitivity analysis of the pain intensity.


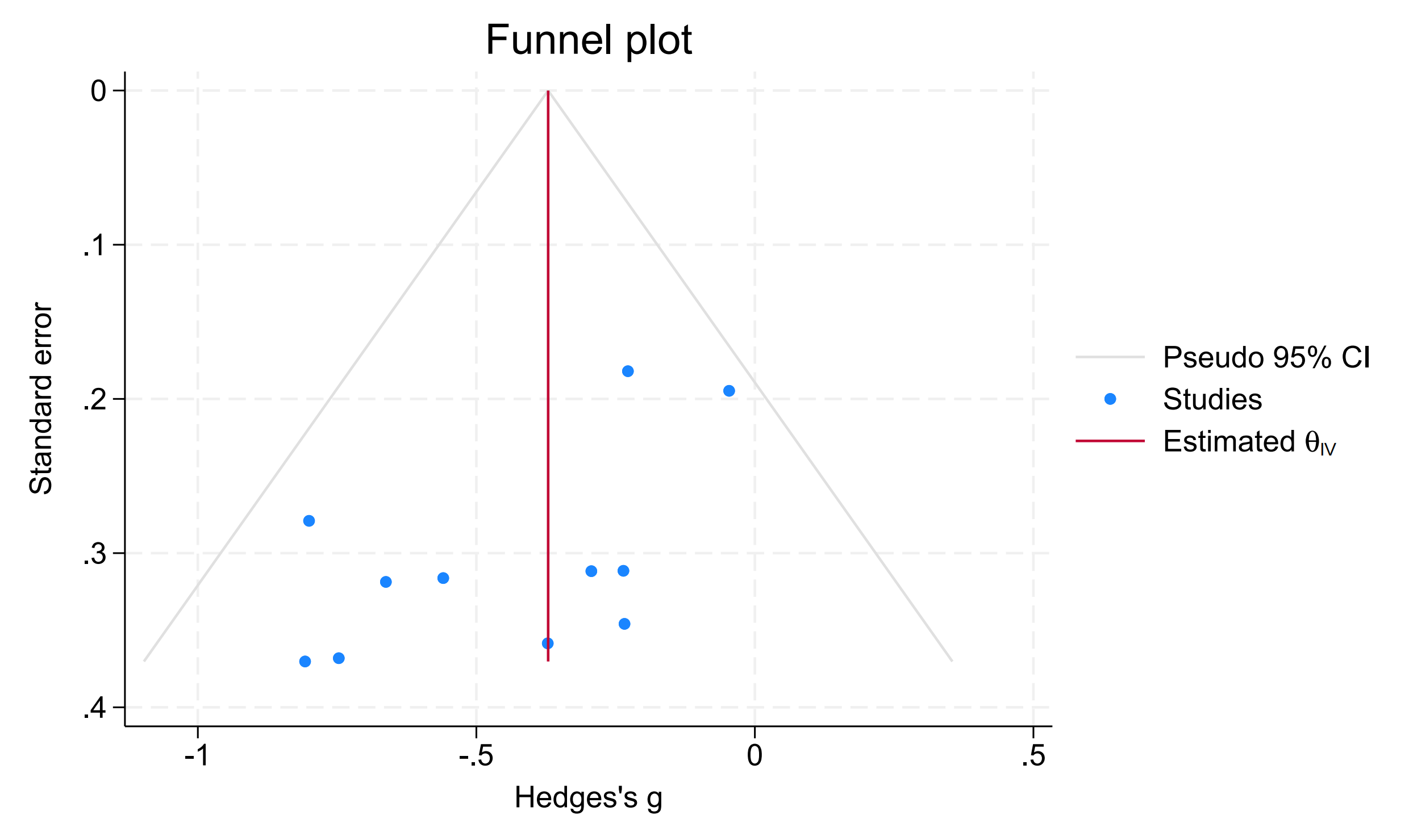


**Figure S5**. Funnel plot of the physical function.

**Figure S6**. Sensitivity analysis of the physical function.


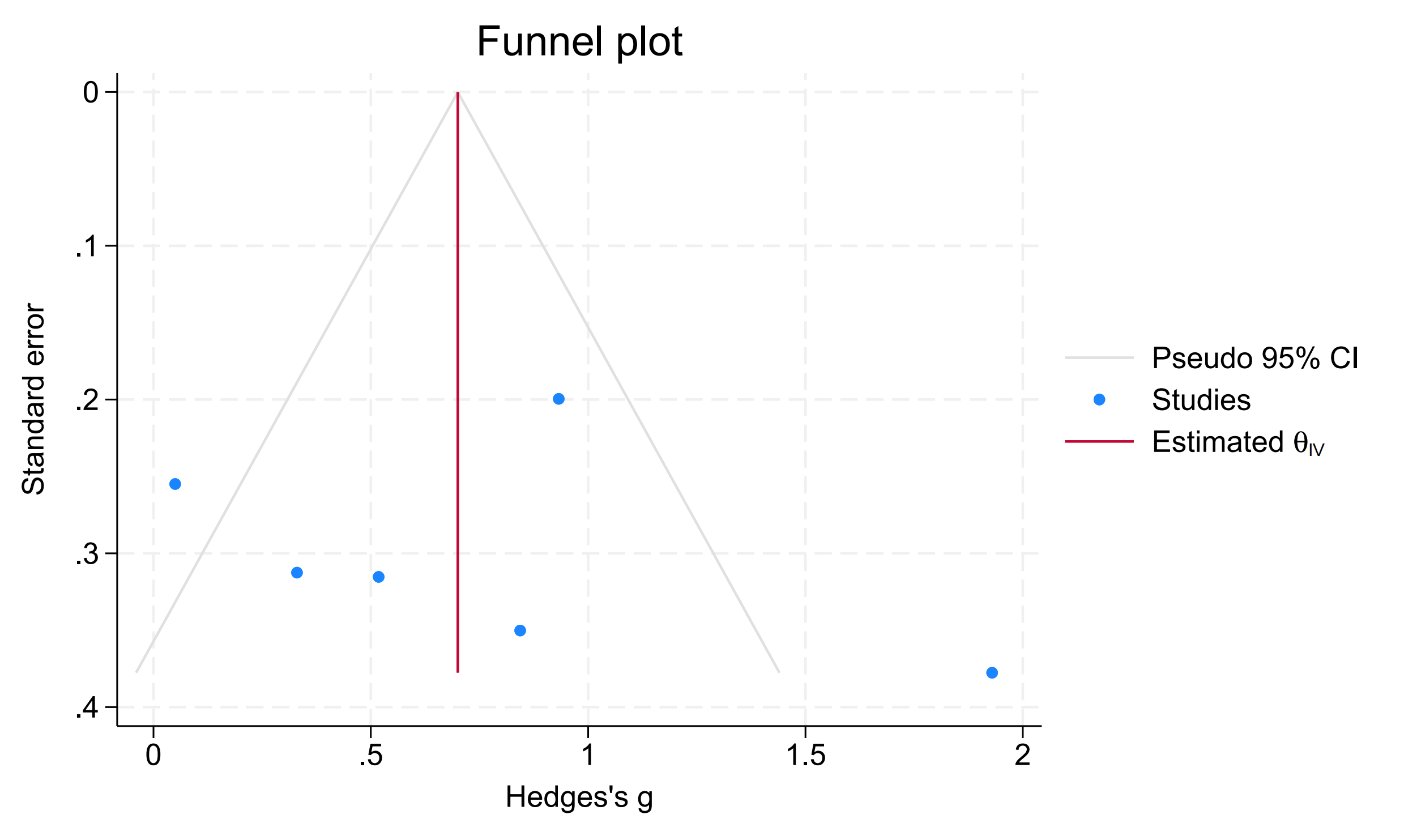


**Figure S7**. Funnel plot of the walk capacity.

**Figure S8**. Sensitivity analysis of the walk capacity.
